# Developing and employing ideal teams for optimal global health outcomes

**DOI:** 10.7189/jogh.11.02001

**Published:** 2021-01-30

**Authors:** Casey Perez, Diana Aguirre, Timothy Clark, James Morgan, Oscar Agueda, Gretchen Thompson, Edwin K Burkett

**Affiliations:** 1 Department of Preventive Medicine and Biostatistics, Uniformed Services University of the Health Sciences, Bethesda, Maryland, USA; 2 Henry M. Jackson Foundation for the Advancement of Military Medicine, Bethesda, Maryland, USA; 3 Aptima, Inc., Woburn, Massachusetts, USA; 4 Physicians for Peace, Norfolk, Virginia, USA; 5 US Army South, Joint Base San Antonio-Fort Sam Houston, Texas, USA; 6 FHI 360, Durham, North Carolina, USA; 7 Defense Institute for Medical Operations, Joint Base San Antonio-Lackland, Texas, USA

Global health engagement (GHE) has been an important aspect of the US military as a security cooperation tool to meet the US Government’s 3D foreign policy strategy (Diplomacy, Development, and Defense) [1]. The US Department of Defense (DoD) has played a critical role in global health by providing expeditionary medical personnel to aid populations in crises. Historically, the DoD has primarily employed non-specific, large-scale direct patient care activities [2] that often present significant challenges to achieving sustainable, ethical, and positive health outcomes and enduring geopolitical gains. GHE activities are typically conducted in areas of a partner nation with limited health sector resources and infrastructure, alongside civilian or military counterparts seeking solutions to significant health needs of the local population [3]. One week long direct care activities have limited mutual benefit and are also difficult to monitor and evaluate due to a focus on process metrics and non-specific health and political impact [2].

Non-governmental organizations (NGOs) are key components in improving global health problems from HIV to family health planning and reproductive health services [4]. Many NGOs understand the importance of building capacity and educating key personnel in local communities in order to make a lasting impact. For example, Physicians for Peace is a NGO that educates and supports local providers to heal their community [5]. They also work alongside local government to address their priority needs and to support gaps in training [5]. Another example is Family Health International (FHI) 360, an organization that is focused on building capacity using research and partnerships to provide efficient solutions for a community’s problems [6]. FHI 360 partners with governments and organizations of the partner nation to bring about health care, education, behavioral changes and improve access to services. NGOs can be a model to generate efficient and sustainable approaches for military GHE activities. Within the development arena, international NGOs often fill the formidable gap between the needs of local populations and the limited capacity of host nation governments and infrastructure to meet these needs. Research has also shown that international NGOs are important actors in building local civil society [7] capable of creating more favorable conditions for security objectives [8].

The Embedded Health Engagement Team (EHET) model is a new joint military concept that shows promise for effectively meeting the 3D strategy. The concept was developed in response to the national security of 2012 Priorities for the 21st Century Defense: “Whenever possible, we will develop innovative, low-cost, and small-footprint approaches to achieve our security objectives” [9]. The EHET model is composed of a small team (fewer than 12 members) of joint US Military personnel that are equipped with the skills, language capability and experience needed for their specified tasks for the host nation [2]. The tasks are based on the nation’s health priorities including, but not limited to, primary care, veterinary medicine, preventive health and environmental health needs. Because of the preparation needed, the mission are of a longer duration (minimum of 2 weeks) and may include intentional recurrent visits of the team members to the same location to foster local ownership and sustainable effects. Recurrent operations build partner capability and interoperability by integrating members within the partner nation’s existing health system. Small, effective teams also lower the costs and bring greater returns on investment. **Figure 1** shows GHE competencies and EHET characteristics. A similar model is already in use by the US Army South- the Medical Training Exercise Program (MEDTEP), consisting of a small team that recurrently visits the same area to build capacity of the local population.

**Figure 1:**
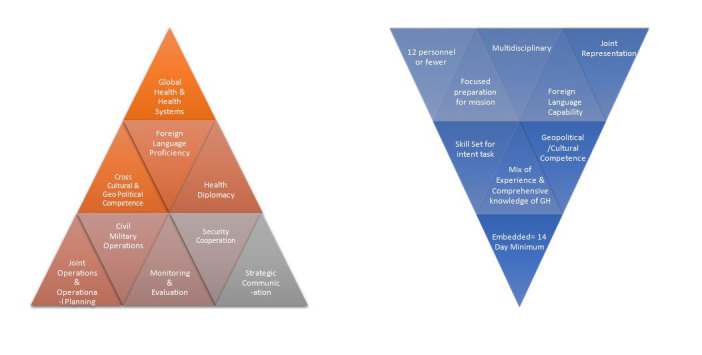
Joint core global health engagement competencies juxtaposed with EHET composition [10]. The base competencies are more strategic in nature with more specificity at apex. EHET capability is an integration of team member capabilities.

Learning from both military and civilian experiences in small team global health activities is required to achieve positive outcomes with partner nations and their health systems. The DoD serves a supporting role to NGOs and civilian government agencies. Therefore, understanding NGO perspectives about EHETs, MEDTEPs and their successes with GHE activities can help achieve a new model for the DoD [11]. A panel of five military and civilian experts were convened at the AMSUS 2019 Annual Meeting. This paper reviews and summarizes key elements of the panel’s experiences with global health teams and their recommendations to significantly improve applied global health and DoD global health engagement.

## APPROACH

The panelists represented civilian NGOs, private companies, and military organizations. They brought expertise in global health activities, surgical care, family planning and reproductive health, data engineering, team evaluation, and Army medical operations. The discussion focused on understanding whether civilian NGOs and military medical capabilities have synergistic thoughts, concepts and successes that can be applied successfully to the DoD’s GHE activities through a small team capability.

The panel discussion was transcribed verbatim and themes were generated from the discussion. Overall, the objective of this paper and the panel discussion was to help participants achieve three objectives: (1) to analyze the concept of ideal embedded health capability teams, (2) to evaluate elements and competencies associated with ideal embedded health capability teams, and (3) to use the knowledge exchanged in the panel to design and employ more effective health engagement teams to achieve improved health and development outcomes in all types of global health scenarios.

## OUTCOMES

Four themes emerged from the panelists while discussing their experiences with GHE activities: (1) building relationships with partners through sustainability and continuity, (2) robust evaluations to enable accurate assertions of performance and impact, (3) evaluating and tailoring team composition, and (4) the need for strategic level engagement. There was an additional discussion about two theories of change to best model the embedded capability teams. **Table 1** provides a summary of the four themes synthesized into recommendations for ideal global health teams.

**Table 1:** Expert panel recommendations for successful global health teams

Key recommendations	Details
1. Building relationships	Embed specialists into host nation’s health care structures.
	Identify host nation’s needs and priorities.
	Enhance health system legitimacy.
	Ensure long-term impact with recurrent visits and continuity
2. Monitoring and evaluation	Passively measure team dynamics - communicate internally and with partner nations.
	Digital platforms can influence data collection compliance.
	Small teams can be more conducive to SMART objectives, data collection and analysis.
	Aggregate data from many missions will reveal more valid and reliable impact.
3. Team composition	Select team members with global health experience and cultural competence.
	Monitoring and evaluation expert to help with evaluation of team performance and data collection.
	Team preparation prior to deployment is critical to learning about the host nation’s needs and resources.
	Small teams can build trust with the host nation and achieve continuity of care and meeting long-term health outcomes.
4. Strategic level engagement	Small teams can effectively flex to meet theater campaign plan/security cooperation objectives, while still supporting the host nation priorities.

### Building relationships: Sustainability and continuity

The panelists discussed the importance of building relationships with the partner nation in order to understand the needs of the local population and its existing health system. The key to meeting health outcomes and program objectives “*is continually building a relationship.*” In order to build relationships, team members emphasized the importance of embedding personnel within the nation’s health system. The following anecdote provides an example of the functions of a nurse from an NGO:

“…[as] part of the multinational collaboration working at the hospital…and she’s physically present…for two weeks, twice a year. But very importantly, her in-person trainings are woven together with weekly communication with a whole cohort of multi-specialty, multidisciplinary trainers from several countries and NGOs. This weekly collaboration came at her initiative, because she said it’s not enough to be there in person two weeks twice a year. We have to continue the relationship and make sure things are happening.”

Another panelist that works in an international development organization expressed collaborating with different national government counterparts such as the ministry of health, ministry of education, or even the nation’s military to build capacity in the countries.

Embedding health specialists within the existing health care structure also ensures sustainability for the partner nation when the team departs. One of the military panelists stated, “*The exchange of knowledge and expertise between professionals would greatly enhance the capacity of the partner nation within their health service*.”

Working with the partner nation in identifying priorities will be best measured by a needs assessment in order to know how to prioritize deployments. Building relationships is also conducive to respecting the nation’s culture and understanding their nation’s health priorities. One of the panelists described his organization’s process:

“After we meet a potential new partner, we start to build our relationship with them. That begins with a joint assessment of their status, their expressed needs and concerns. Then based on this assessment we determine, if and how, we can match our resources - and that’s especially our volunteer trainers - to meet the partner’s needs.”

Ensuring long term engagements helps foster relationships and aligns with the DoD’s four-year integrated country strategies and the Combatant Command’s five-year country plans. The military expert panelist described how his team visits the partner nation “*every six months to the same area. This will permit a continuity of care … that we are trying to do is assist the population and so for them to be able to develop a better program and a sustainable program in their county or in their location.*” The MEDTEP model involves three- to five-year engagements (every six months), instead of the traditional GHE model of returning every five years.

### Evaluation: Assertions of performance and impact

The second theme that emerged from the panel discussion was the critical component of robust evaluation during the GHE activities. There is currently a lack of evidence and minimal measurement of the effectiveness and impact of GHE activities. The corresponding author reviewed 414 records and did not find any evidence of “*intentional preparation of any of the personnel who went on these 414 missions*” [11].

One key challenge with GHE evaluation processes is how to best measure effectiveness in a non-intrusive way. The data engineer panelist stated that innovations in evaluation should focus on “*passively measuring these team dynamics and really get us some of the information about how these teams perform together*.” The panelist also suggested employing methods and technologies to generate data about how these teams communicate and engage both internally and with partner nations. The discussion also highlighted methods that can provide an ability to evaluate a team’s level of cohesion by measuring communication density and the network structures of communication. It also enables follow-on analytics that can determine the optimal team composition and even the best leadership structure for the team. Evaluating the team in this way makes it easier to find deficiencies that can be addressed in training.

Another panelist described their experience with evaluation and how they collect data during GHE activities. She reported having monitoring and evaluation (M&E) experts as part of their team to track their activities, outputs, progress, and deliverables. The collected data can paint a realistic picture on the team’s impact and evaluation. The panelist also described the team having a digital data collection process and mobile data collection that helps immensely in the field and influences data collection compliance. Finally, the panelist indicated that effective evaluation for GHE activities should include both a process and outcome evaluation – measuring not only outputs from the activity but ultimately the impact.

### Team composition

A third theme determined critical to achieving GHE mission objectives is the selection of team members. Similar processes were identified across panelists and their organizations. Program success depends on selecting and deploying team members with experience and cultural competence. Having expertise in the field, of the partner nation’s culture and needs emerged as efficient in collaborating together as a team with the partner nation. A panelist also described having an M&E expert included in the team to help with evaluation of team performance and data collection.

The panelists highlighted that preparing team members prior to deployment is critical to learning about the partner nation’s needs and the resources needed for the project. One of the panelists described the process:

“The construct of the team is dependent on what the needs of the area, location, the partner nation and the security cooperation office from the U.S. teams decides what needs to be. Once this is done, an assessment is done in the area and then the team is put together.”

In particular, a small team’s engagement strategy was recommended as the best model for building trust with the partner nation and achieving long-term change on health outcomes. A panelist described scenarios where four or five members deployed together to help with burn care rehab training in the nation or embedding a nurse in a local hospital for multiple years to support education initiatives. Selecting a small number of subject matter experts helps with continuity of care and meeting health outcomes. An audience member put forth an idea about the importance of monitoring the team in terms of stress level and communication dynamics and adjusting the team composition, as necessary.

### Strategic level engagement

Theater Security strategy emphasizes building interoperable and sustainable partnerships and partner capacity. The panelists indicated that selecting small teams can support effective strategic principles through tailorable capabilities to meet the Theater Strategy and Security Cooperation objectives, while still emphasizing the need to support host nation priorities [12]. In order to bring about change, the Diffusion of Innovations model [13] is effective for instituting embedded teams as an optimal capability for the military and other health practitioners. The Explanatory model when employed in small teams will generate change and improvement within international communities [14].

Using the principles of the Diffusion model, the innovation of deploying small, embedded capability teams spreads by communicating over time amongst members of the DoD [13]. Embedded capability teams are a paradigm breaking innovation that should be adapted for global health activities. The more effective the innovation, the faster it is adopted. The rate of adoption of the new idea is determined by the relative advantage (if individuals perceive the innovation as advantageous), compatibility (perceive the innovation as being uniform with existing values and needs of adopters), complexity (perceive the innovation as difficult to understand and use), trialability (degree to which an innovation may be experimented), and observability (results of an innovation are visible to others) [13]. Previous studies identified a fast approach of diffusion when communicating innovations to leaders in the community that are able to encourage and change behaviors of their peers [15]. Several geographical combatant commanders and component commanders (SOUTHCOM, AFSOUTH, ARSOUTH) have adopted the embedded capability teams and can encourage others with adoption.

The Explanatory model emphasizes culture in reflecting meaning to the patient or community [14]. Embedded capability teams allow cultural context to enhance relationships with the partner nation. Collaborating with the partner nation helps in determining the meaning of the effort for both sides and contextualize interpretations of the effects and decisions leading to adjustments and successful efforts. One of the panelists stated, “*What we think is critical to success is to respect cultural differences and resource limitations, and to consider locally identified priorities*.” Allowing the partner nation to determine their priority needs supports a community-based approaches and long-term capacity building.

## MEETING DISCUSSIONS

This paper summarizes a panel discussion focused on learning from the past experiences of civilian and military personnel and organizations to identify innovations and new best practices for GHE initiatives. The discussion revealed a consensus of methods and goals to achieving positive GHE outcomes, with broad agreement about the importance of developing and maintaining productive and mutually-beneficial relationships with partner nations. The civilian expert panelists confirmed that fostering relationships with a partner nation’s stakeholders can create lasting change, and that embedding specialists into the existing health care structures during long-term engagements helps build capacity for partner nations. Initial research of a pilot test of EHET in Costa Rica and MEDTEP in Guatemala showed that working alongside the host clinic’s staff was beneficial to both entities; however, more non-clinical embedded capability teams are needed to build capacity, such as surgery, disaster response, and administration [2].

The panel discussion suggested the important role that M&E plays in both achieving and validating positive outcomes. Panelists provided examples ranging from collecting digital data for impact analysis to new methods for evaluating team performance and deliverables that are imperative to informing the capability of the team. Meticulously tracking GHE activities over time through shared outcome measures with partner nations provides robust time series data that can emphasize an effort’s impact [16]. Evaluating teams should also include SMART (Specific, Measurable, Attainable, Reliable, and Timely) objectives that are selected prior to deployment to create easier data collection, especially among smaller teams. Lastly, the evaluation should be informed by a sound logic model and theory of change, which explains the desired impact of the activity being implemented.

The majority of the panelists pointed to the importance of team composition, and recommended methods for putting together a team. Team composition should focus on being responsive to the partner nation’s needs, which can be achieved through activities such as a detailed needs assessments with partner nations prior to deployment. A small, multidisciplinary team with previous GHE activity experience helps foster relationships and exchange knowledge to build collaborative plans with the partner nation. Small, tailored teams are also likely to develop trust and earn mutual respect to aid in the partner nation’s problems [12].

## CONCLUSION

The panelists agreed that embedding teams within a partner nation’s health system or community is more effective than a preemptive insert of an existing operational plan. Building sustainable strategic level partnerships increases the likelihood of effective support for Theater Security Cooperation goals. Small teams are better suited to meet Theater Campaign Plans since they can be tailored and embedded within a partner nation’s existing systems to build partnerships and capability.

Overall, the panelists conveyed four major objectives needed to improve global health outcomes. From the panelists’ experience, smaller teams with experienced, culturally competent, multidisciplinary members are best suited to the projects’ objectives. Finally, the evaluation of teams is critical to achieving short-term and long-term outcomes.
